# A case report of Bart syndrome

**DOI:** 10.1002/ccr3.7612

**Published:** 2023-06-26

**Authors:** Seyed Amirabbas Sharif, Alieh Mohammadzadeh, Mohammad Mahdi Heidari, Rasoul Etesam Por

**Affiliations:** ^1^ Department of Pediatric Kashan University of Medical Sciences Kashan Iran; ^2^ Student Research Committee Kashan University of Medical Sciences Kashan Iran

**Keywords:** aplasia cutis, Bart syndrome, epidermolysis bullosa, genetic skin disorder, newborn

## Abstract

Bart syndrome is a rare condition characterized by epidermolysis bullosa (EB), aplasia cutis (AC), and nail abnormalities. Aplasia cutis congenita type VI was first described in 1966 by Bart et al. This article reports a case of Bart syndrome with ear malformation in a male Afghan newborn. To the authors' knowledge, this is the first case of Bart syndrome reported in an Afghan family.

## Graphical abstract

Figure 1 in terms of appearance and clinical and Figure number 3 in terms of pathology will help a lot in diagnosing the disease.

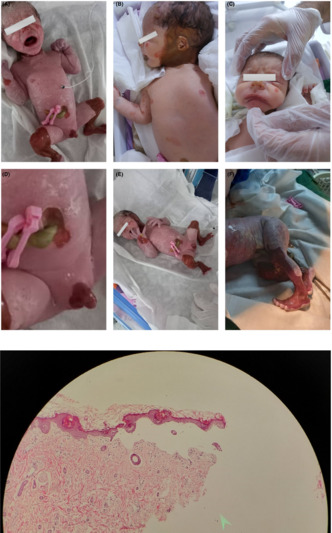

## 1 CASE PRESENTATION

**FIGURE 1:**
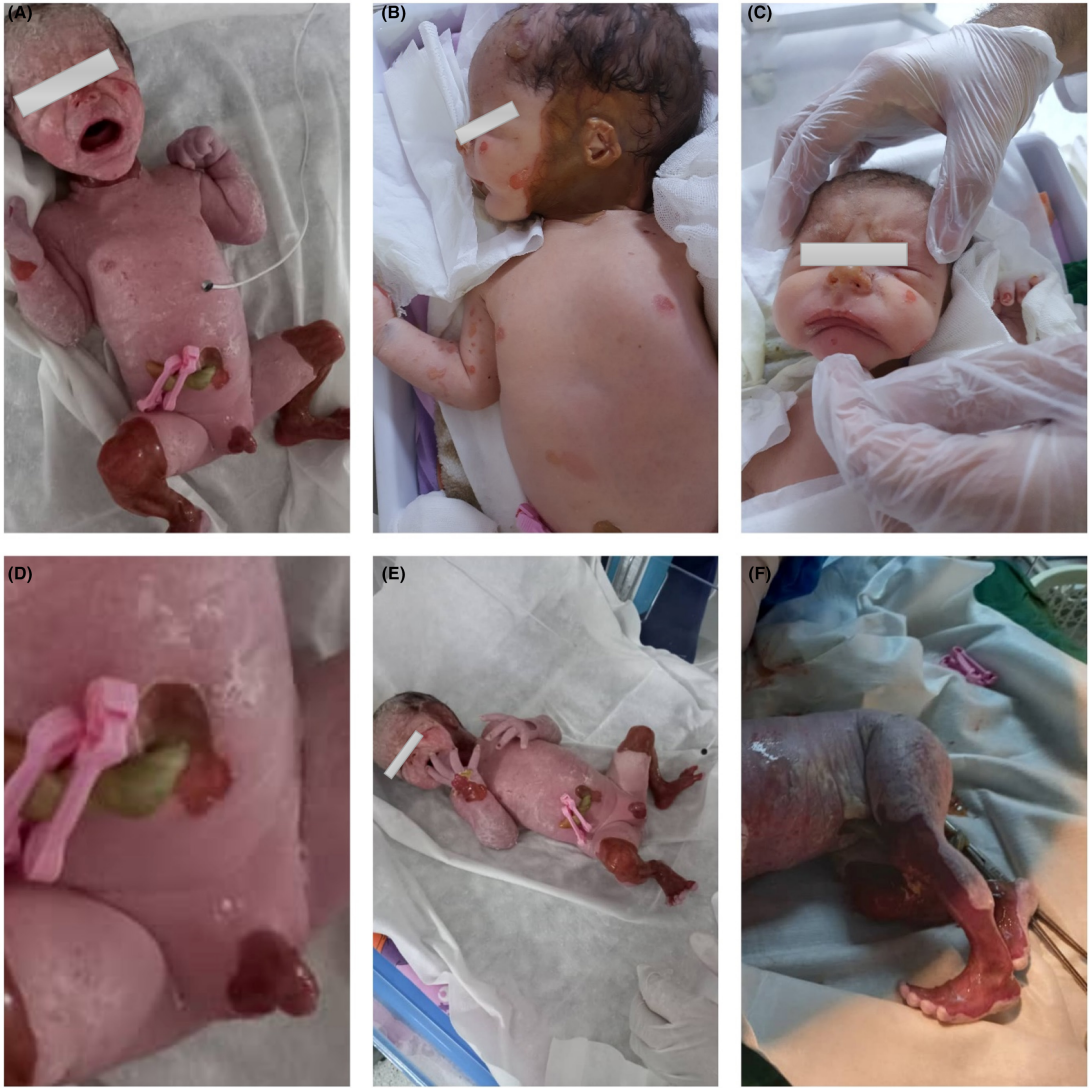
Lack of skin on the anteromedial part of the shin (A), anterolateral part of the neck that surrounding the periauricular (B), the tip of the nose (C), around the penis and periumbilical (D), and the dorsum of both hands and feet (E, F).

At 7:21 p.m. on July 27, a male term newborn (with a gestational age of 36 weeks and 4 days) was referred to the neonatal intensive care unit of Shahid Beheshti Hospital in Kashan, Iran. The newborn was delivered via caesarian to a 32‐year‐old multigravida mother. Despite having normal Apgar scores, the infant was brought to the newborn critical care unit due to skin lesion. The mother denied having been exposed to drugs or radiation throughout her pregnancy. The phrase “High risk” was recorded in the quad marker screening for the baby's mother, and minor ectasia of two kidneys and echogenic intracardiac focus was detected in the anomaly scan on March 7, 2021. Amniocentesis was conducted on her 3 days later, which proved normal. On April 10, 2021, echocardiography was also performed for her fetus, and the left ventricular echogenic center reported in the first time was normal in the next echo. The parents were not relatives. There was no history of such problems or skin disorders in the family. An examination revealed the lack of skin on the anteromedial part of the shin (Figure 1A), the anterolateral aspect of the neck, periauricular (Figure 1B), the tip of the nose, and nail (Figure 1C), around the penis, periumbilical region (Figure 1D), and dorsum part of both hands and feet (Figure 1E,F).

**FIGURE 2:**
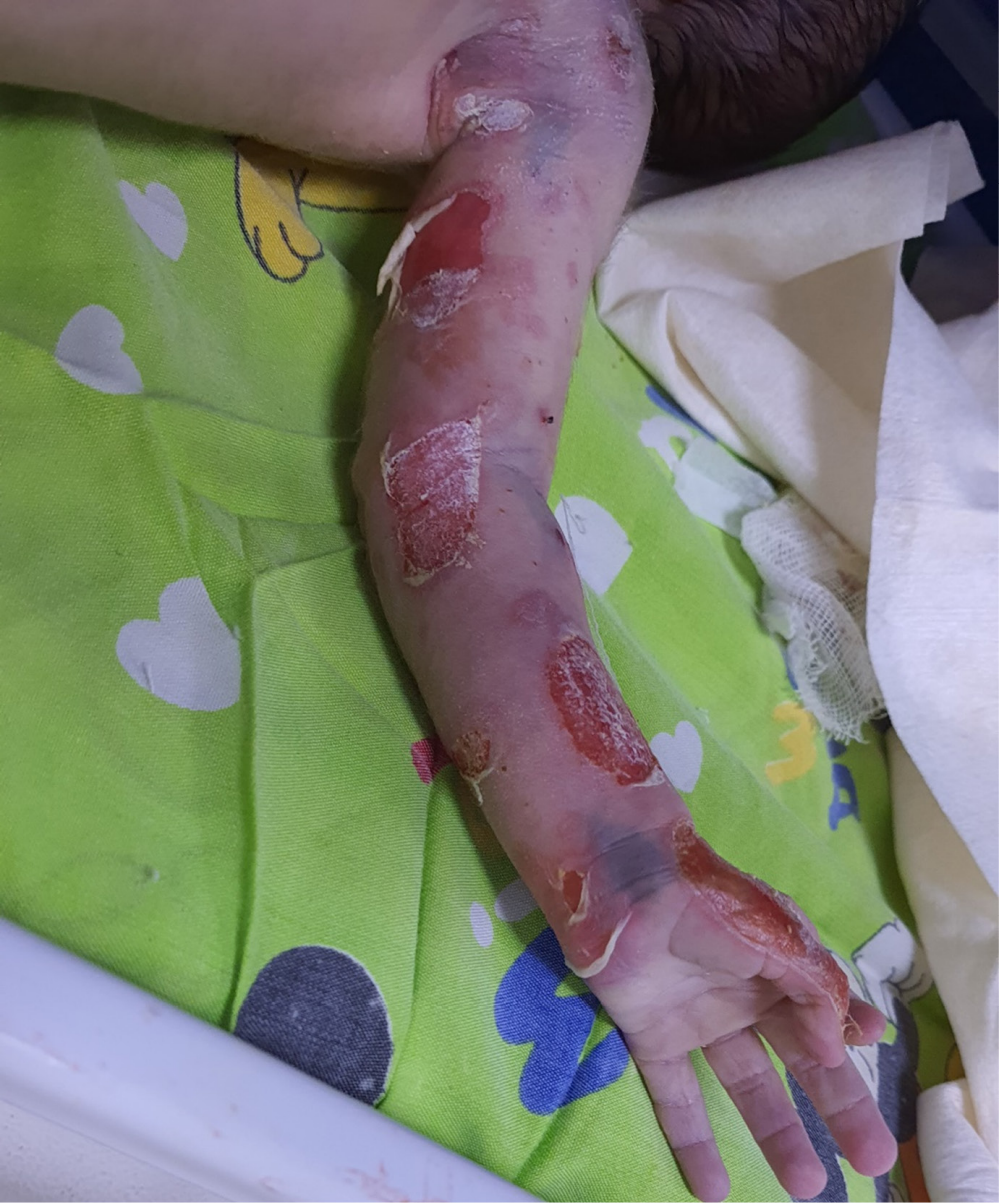
Blisters and erosion on the inner aspect of the left hand.

The scalp and mucous membranes were spared. The overall checkup was normal. The infant developed blisters over the previous lesions after 2 days. Blistering also appeared over normal skin in response to minor trauma or friction (Figure 2).

Several laboratory tests were performed; the baby's complete blood count, electrolytes, and liver and renal function tests were all within normal limits; however, after 2 days, CRP, creatinine, and urea levels increased, prompting us to change our antimicrobial regimen.

Electrolyte disturbances (hyperkalemia, hypernatremia) developed due to the lack of iv‐line and maintenance fluid due to the fasting condition. According to the baby's condition, a request was made for the placement of an umbilical catheter for the patient by the general surgery service. The patient went into cardiac arrest during the procedure. He was successfully resuscitated and transferred to NICU. He was extubated the following day. His electrolyte disturbance resolved on placement of umbilical catheter and diuresis was established. After that, the patient was treated with one and a half times of the maintenance fluid serum, and his electrolyte disturbances were resolved, and diuresis was established.

He was referred for dermatology, cardiology, and gastrointestinal consultation. Due to the emergence of oral lesions, the dermatologist administered nystatin solution in the amount of 0.5 cc every 6 hours, continued topical mupirocin, and a new antimicrobial regimen. The echocardiogram results were normal.

**FIGURE 3:**
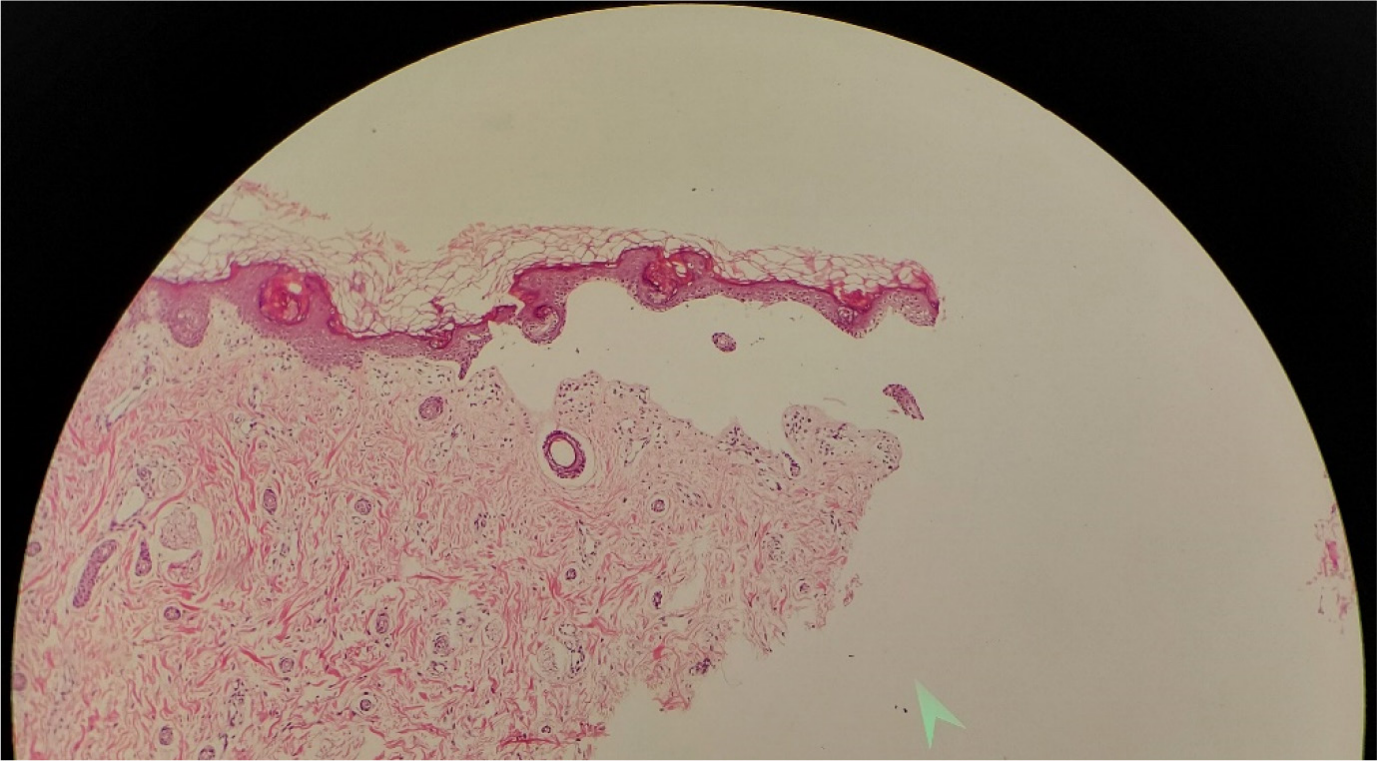
Subepidermal blister development, consistent with epidermolysis bullosa.

A region with blister and normal skin on the left arm was used to get a skin biopsy. A histopathological examination of the skin biopsy revealed subepidermal blister development, consistent with epidermolysis bullosa (Figure 3). Immunofluorescence staining and electron microscopy were not available in our hospital. However, samples were sent to Razi Hospital, Tehran, Iran, and after 11 days pathologist commented that: the DIF conclusion in negative but correlation with clinical findings is recommended.

We treated the patient's body lesions with daily dressings of Vaseline gauze and gauze saturated with normal saline and a twice‐day application of topical mupirocin cream.

As the neonate died at Day 5 of life; due to severe neonatal sepsis. The disease's rapid progression and fatal course interrupted the planned ultrastructural and genetic linkage investigations.

## 2 DISCUSSION

Bart syndrome, which was first described by Bart et al. nearly half a century ago, is a kind of aplasia cutis congenita (ACC).^1^, ^2^ ACC is a rare heterogeneous disorder marked by a localized loss of skin.^3^ Other congenital malformations that can occur in more severe cases of Bart syndrome, particularly those linked with junctional epidermolysis bullosa, include pyloric atresia, ureteral stenosis, renal abnormalities, rudimentary ear development, flattened nose, large nasal root, and wide‐set eyes.^4^, ^5^ In our case, there were no associated anomalies.

The inheritance pattern of Bart syndrome seems to be autosomal dominant but sporadic case had been reported.^6^ Because there was no consanguinity between the patient's parents or a family history of a comparable lesion, our case was sporadic. The clinical appearance of Bart syndrome is usually used to make a diagnosis. In some case, a skin biopsy will be required to determine the type of epidermolysis bullosa and a genetic testing to determine the particular gene mutation, which may allow us to confirm the final diagnosis.^7^ The diagnosis of Bart syndrome was made in our case based on the typical clinical presentation, including localized lack of skin across the anteromedial aspect of both lower legs, blistering of the skin, nail degeneration, and information from a skin biopsy (histopathology and direct immunofluorescence). Bart syndrome treatment options range from conservative to secondary intention to surgical intervention.^8^ Similar to omran et al., there were skin lesions in our case, but there was no ear malformation in our newborn; omran et al. reported a case of Barrett's syndrome with well‐demarcated noninflammatory skin defects covered by a red thin translucent membrane localized to the nose, around both wrist joints, the front of the left knee, dorsum of both feet, around both ankle joints and around the lower third of the legs, and ear malformation in an Egyptian newborn.^9^

In this case, we treated the patient's body lesions with daily dressings of Vaseline gauze and gauze saturated with normal saline, as well as twice‐day application of topical mupirocin cream. But Alfayez et al. performed conservative wound care with topical antibacterial cream (fusidic acid cream 2%) applied twice per day and nonadhesive dressing^10^; this difference in the method of supportive treatment may be effective in the final outcome of the disease.

Many factors influence the prognosis of Bart syndrome, including the degree and extent of ACC, related abnormalities, and therapeutic success.^6^, ^11^ Close follow‐up is required for significant consequences such as infection, hypothermia, hemorrhage and hypoglycemia. Except for infection, no major problems were observed in our instance as of the writing of this study.

As the neonate died on Day 5 of life, due to severe neonatal sepsis, the case reported in our study had a similar fate to the case reported by omran et al.,^9^ but Alfayez et al. stated that after 1 week, the infant was discharged, and the mother was given detailed instructions about handling the baby and continuing local wound care.^10^ This issue determines the importance of the type of supportive treatment and the severity of the deterioration of the patient's condition in the prognosis of the disease, but what is clear is the importance of early treatment for all neonates.

## 3 CONCLUSION

Bart syndrome is an uncommon but significant disease because, if it happens in a family, there is a chance that future children may have the same problem, assuming that it is genetic. Commonly, the syndrome has a good prognosis, but it should be managed as early as possible to reach the best outcome. The available treatments are primarily supportive, and in the absence of serious complications, the patients will have a favorable prognosis, and with appropriate follow‐up periods, these patients will have the option of living.

## AUTHOR CONTRIBUTIONS

**Seyed Amirabbas Sharif:** Investigation; supervision. **Aliyeh Mohammadzadeh:** Investigation; supervision. **Mohammad Mahdi Heidari:** Project administration; writing – original draft. **Rasoul Etesam Por:** Writing – review and editing.

## FUNDING INFORMATION

This study was financially supported by Kashan Medical Sciences University, Isfahan, center Iran.

## CONFLICT OF INTEREST STATEMENT

The authors have no conflict of interest to declare.

## ETHICS STATEMENT

This study was reviewed and approved by the Ethics Committee of Kashan University of Medical Sciences (IR.KAUMS.MEDNT.REC.1401.017), and all participants gave written informed consent.

## CONSENT

Written informed consent was obtained from the patient to publish this report in accordance with the journal's patient consent policy.

## Data Availability

The data that support the findings of this study are available on request from the corresponding author. The data are not publicly available due to privacy or ethical restrictions.
